# The long-term outcomes of epilepsy surgery

**DOI:** 10.1371/journal.pone.0196274

**Published:** 2018-05-16

**Authors:** Midhun Mohan, Simon Keller, Andrew Nicolson, Shubhabrata Biswas, David Smith, Jibril Osman Farah, Paul Eldridge, Udo Wieshmann

**Affiliations:** 1 The Walton Centre, NHS Foundation Trust, Liverpool, Merseyside, United Kingdom; 2 Department of Molecular and Clinical Pharmacology, Institute of Translational Medicine, University of Liverpool, Liverpool, Merseyside, United Kingdom; University of Modena and Reggio Emilia, ITALY

## Abstract

**Objective:**

Despite modern anti-epileptic drug treatment, approximately 30% of epilepsies remain medically refractory and for these patients, epilepsy surgery may be a treatment option. There have been numerous studies demonstrating good outcome of epilepsy surgery in the short to median term however, there are a limited number of studies looking at the long-term outcomes. The aim of this study was to ascertain the long-term outcome of resective epilepsy surgery in a large neurosurgery hospital in the U.K.

**Methods:**

This a retrospective analysis of prospectively collected data. We used the 2001 International League Against Epilepsy (ILAE) classification system to classify seizure freedom and Kaplan-Meier survival analysis to estimate the probability of seizure freedom.

**Results:**

We included 284 patients who underwent epilepsy surgery (178 anterior temporal lobe resections, 37 selective amygdalohippocampectomies, 33 temporal lesionectomies, 36 extratemporal lesionectomies), and had a prospective median follow-up of 5 years (range 1–27). Kaplan-Meier estimates showed that 47% (95% CI 40–58) remained seizure free (apart from simple partial seizures) at 5 years and 38% (95% CI 31–45) at 10 years after surgery. 74% (95% CI 69–80) had a greater than 50% seizure reduction at 5 years and 70% (95% CI 64–77) at 10 years. Patients who had an amygdalohippocampectomy were more likely to have seizure recurrence than patients who had an anterior temporal lobe resection (p = 0.006) and temporal lesionectomy (p = 0.029). There was no significant difference between extra temporal and temporal lesionectomies. Hippocampal sclerosis was associated with a good outcome but declined in relative frequency over the years.

**Conclusion:**

The vast majority of patients who were not seizure free experienced at least a substantial and long-lasting reduction in seizure frequency. A positive long-term outcome after epilepsy surgery is possible for many patients and especially those with hippocampal sclerosis or those who had anterior temporal lobe resections.

## Introduction

Epilepsy is the most common serious neurological disorder with medical treatment failing to control seizures in up to 30% of patients [1]. Temporal lobe epilepsy (TLE) is the most common pharmacoresistant focal epilepsy disorder [2,3] and thus, temporal lobe resections are the most common operations done in adults with epilepsy, and has class I evidence for its short-term efficacy compared to continuing medical treatment [4,5]. Other operations done for epilepsy include extratemporal resection, [6] and hemispherectomy [7] to name a few, however there are several other operations that can be performed with their indications being multi-factorial. Surgery can be extremely effective for patients with TLE, significantly reducing seizure frequency and improving quality of life in the majority of patients. However, there are contrasting reports regarding the proportion of patients attaining seizure freedom after temporal lobe surgery for refractory epilepsy, which may range from 35–80%[4,8–12]. The length of time to follow up assessment after surgery and whether seizure freedom is defined as freedom from all kinds of seizures and aura or disabling seizures only, are the factors that likely explain the previously reported differences in postsurgical seizure outcomes after short follow up periods [13].

In the only randomised controlled trial of surgery for refractory TLE, it was reported that 58% of patients were free from seizures impairing awareness and 38% were free from any seizure related symptom one year after surgery [4]. This contrasted to 8% who became seizure-free in the non-surgical control group. Given that the majority of patients who are potential candidates for surgery are relatively young adults, long-term postoperative seizure freedom data is crucial and arguably more significant than short-term outcomes. Long-term outcome data is also necessary so that patients can make an informed decision regarding invasive neurosurgery from a risk-benefit perspective. However, long-term outcomes of epilepsy surgery are less frequently documented and seizure freedom rates range from 21% - 91% at 5 years [9,12,14–17] and 41% - 81% at 10 years [9,12,16,18]. Several studies have been published looking at predictors of both short-term and long-term outcomes after epilepsy surgery. The predictors of good short-term postoperative seizure freedom include: complete surgical resection, presence of hippocampal sclerosis, MRI positive (abnormal pre-operative MRI), no intracranial monitoring being performed, concordance with EEG and pre-operative MRI, no evidence of focal cortical dysplasia, no evidence of malformation of cortical development, a history of febrile seizures, presence of a tumour, unilateral interictal spikes and a right sided resection [19–21]. The predictors of bad postoperative seizure freedom include: long duration of epilepsy prior to surgery [18,22–26], higher age at surgery [9,16,27,28], high seizure frequency at baseline (pre-surgery) [22,29], generalised convulsive seizures at baseline (pre-surgery) [12,27,30], early postoperative seizures [31–33] and postoperative interictal epileptiform discharges [34–36]. However it is important to note there are some studies that found no predictors even after using multivariate analysis approaches [14,37,38].

There is currently a paucity in the literature of prospective studies investigating long-term outcomes after epilepsy surgery, therefore the primary aim of this study was to ascertain long-term postoperative seizure freedom rates at a large neurosurgery hospital in the United Kingdom.

## Materials and methods

### Overview and ethics

The study was approved by the clinical audit group at the Walton Centre NHS Foundation Trust, Liverpool who classified the work as a service evaluation and thus the study was exempt from requiring individual patient consent and did not require ethics approval. The Walton Centre is a tertiary referral centre covering a catchment area of approximately 3 million people in the North West of England and additionally treats people with refractory epilepsy referred from Northern Ireland, Wales and Yorkshire.

### Study subjects and inclusion criteria

Patients with medically refractory focal epilepsy who had undergone epilepsy surgery were included in the present study. All patients who were offered surgery had epilepsy that failed to respond to at least two anti-epileptic drugs for a minimum of two years.

### Presurgical evaluation

All patients underwent comprehensive standardised presurgical evaluation including documentation of seizure semiology, scalp electroencephalography (EEG), video EEG telemetry, high resolution magnetic resonance imaging (MRI) and neuropsychological evaluation. From the year 1996, all patients had dedicated MRI scans with coronal gradient echo T1 sequence with continuous slices in the highest available resolution and T2 imaging perpendicular to the longitudinal axis of the hippocampus. All MRI negative patients, and patients in whom there was a concern regarding the concordance of non-invasive results had invasive EEG investigations. Until the year 2000, foramen ovale electrodes were typically used [39,40]. After 2000 we used bitemporal depth electrodes. Grid and strip electrodes were implanted if required. Selected patients had the sodium amytal procedure (Wada test) and single photon emission computed tomography (SPECT).

### Operative details

Epilepsy surgery was performed by three functional neurosurgeons (PE, TV, JF) who specialised in the surgical treatment of epilepsy. Operations were performed between 1971 and 2014. In patients with non-dominant (usually right) temporal lobe epilepsy, a standard en-bloc resection was performed. In dominant (usually left) temporal lobe epilepsy a selective amygdalohippocampectomy (AHE) was performed by the trans-middle temporal gyrus approach as described by Niemeyer [41], but with the use of image guidance. Hippocampal head, and at least a part of hippocampal body and the amygdala were removed. The aim was a selective operation leaving other mesiotemporal structures including the parahippocampal gyrus intact. Later in the series, because of a perception that the selective operation had a poorer seizure outcome, the practice was changed to a standard lobe resection. Some of the patients who had relapsed following the selective procedure were offered a standard temporal lobe resection as a second procedure.

All patients had a post-operative MRI which confirmed adequate removal of the intended target. We did not measure the extent of the resection or the volume. However, the limited resections in our case of amygdalohippocampectomy were less successful than standard temporal lobe resections. All patients who had seizure recurrence had MRI scanning to confirm the extent of the resection. One of three neurosurgeons experienced in epilepsy surgery resected a maximum of 6.0cm to 6.5cm of the anterior lateral non-dominant temporal lobe or 4.0cm to 4.5cm of the dominant temporal lobe. The mesial resection included the amygdala and, at a minimum, the anterior 1.0cm to 3.0cm of the hippocampus. Patients undergoing selective an amygdalohippocampectomy had only a very limited resection of the amygdala and the hippocampal head leaving the parahippocampal gyrus intact.

All patients remained on antiepileptic drugs unless they had signs of intoxication for at least 2 years following surgery. Following this, the dose was slowly reduced to achieve a low dose monotherapy. All patients apart from one remained on anti-epileptic drugs in the long term.

### Outcome measures and follow up

This study is a retrospective analysis of prospectively collected data. Patients were followed up prospectively at three months after surgery and then between six and 12 months. Patients who were referred from outside the catchment area were followed up by the local hospitals 12 months after surgery. Long-term outcomes were ascertained from the case notes, the electronic record system of the hospital and from general practitioners. We used the International League Against Epilepsy (ILAE) [42] outcome classification scale. The ILAE outcome scale includes six classes of outcome: Class 1 = seizure free; Class 2 = auras only; Class 3 = one to three seizure days/year and auras; Class 4 = four seizure days/year to 50% reduction in baseline and auras; Class 5 = <50% reduction in baseline number of seizure days to 100% increase in baseline number of seizure days and auras; Class 6 = >100% increase in baseline number of seizure days and auras. Other clinical data collected included age, sex, age of onset of epilepsy, duration of epilepsy, pre-operative MRI diagnosis and surgical approach. We looked at the effect of structural abnormalities as detected by pre-operative MRI rather than post-operative histology since pre-operative MRI is more clinically useful. All authors had access to patient identifiable information and all data was stored locally at the hospital in a secure database on password protected computers.

### Statistical analysis

We used Kaplan-Meier survival analysis[43] to estimate the chance to be free of seizures with loss of awareness (ILAE Class 1 and 2) at various timepoints after surgery. This particular statistical technique was used to address the issue of patients in the study having different lengths of follow up. Patients reach either the endpoint (i.e. seizure recurrence) or are "censored" and the "survival probability" estimated with the remaining "numbers at risk" for a given time point. Kaplan-Meier estimates are used routinely in high impact studies to estimate survival probability and was used in the Lancet publication on the topic of outcomes after epilepsy surgery by de Tisi et al[44].

To ascertain clinical significance, we used the Log Rank (Mantel-Cox) test. This nonparametric test is commonly used to compare survival distributions and when censoring of data is involved. A p-value of less than or equal to 0.05 was deemed as statistically significant.

## Results

### Demographics

From a database containing 897 patients who were discussed at epilepsy surgery multidisciplinary team meetings, we identified 291 patients who had epilepsy surgery. Seven patients were excluded due to incomplete data leaving 284 patients for the study. 143 (50.4%) patients were male, the mean age of onset of epilepsy was 13.7 years (SD 9.91 years) and the mean duration of epilepsy was 19.6 years (SD 11.0 years). All patients were receiving four anti-epileptic drugs (AEDs) prior to surgery and were receiving at least two AEDs at the time of surgery. The mean age when operated on was 33.0 years (SD 10.6 years). 178 (62.7%) patients had an anterior temporal lobe resection (ATL), 37 (13.0%) had an amygdalohippocampectomy (AHE), 33 (11.6%) had a temporal lesionectomy (TLesx), and 36 (12.7%) had an extra temporal lesionectomy (ETLesx). There were no perioperative deaths. One patient had a hemiparesis after a selective amygdalohippocampectomy. Fifteen patients died during the follow up period. The underlying cause of death was sudden unexpected death in epilepsy (SUDEP) in three patients and for the remaining twelve patients the underlying cause of death was unknown. The median duration of follow up was 5 years with a range from 1 to 27.0 years and a total of 1957 person-years. More than half of all surgeries performed were for hippocampal sclerosis (62.6%) and 14.0% were due to tumour: usually low grade gliomas and dysembryoplastic neuroepithelial tumours. There were two patients with meningiomas. The most common vascular abnormality were cavernous haemangiomas, there was only one patient with an arteriovenous malformation in this group. All malformations of cortical development were focal cortical dysplasias type II. MRI diagnosis was confirmed by post-operative histology. In MRI negative patients who had temporal lobe resections all had gliosis on post-operative histology. Table 1 shows pre-operative MRI findings and the operative procedure. For 49 patients, the pre-operative MRI finding was unknown or had various other pathologies (e.g. cystic lesion, middle cerebral artery infarct, meningeal fibrosis) and thus, were excluded from this particular analysis (see S1 Table for further details).

**Table 1:** Pre-operative MRI findings and the operative procedure.

	AHE	ATL	ETLesx	TLesx	Total
**Hippocampal Sclerosis**	33 (22.4%)	114 (77.6%)	0	0	147
**MCD**	0	5 (41.7%)	5 (41.7%)	2 (16.7%)	12
**MRI Negative**	0	19 (95.0%)	0	1 (5.0%)	20
**Tumour**	1 (3.0%)	1 (3.0%)	13 (39.4%)	18 (54.5%)	33
**Vascular**	0	1 (4.3%)	15 (65.2%)	7 (30.4%)	23
**Total**	34	140	33	28	235

AHE = amygdalohippocampectomy, ATL = anterior temporal lobe resection, ETLesx = extra temporal lesionectomy, TLesx = temporal lesionectomy, MCD = malformations of cortical development.

### Seizure freedom

**Fig 1:**
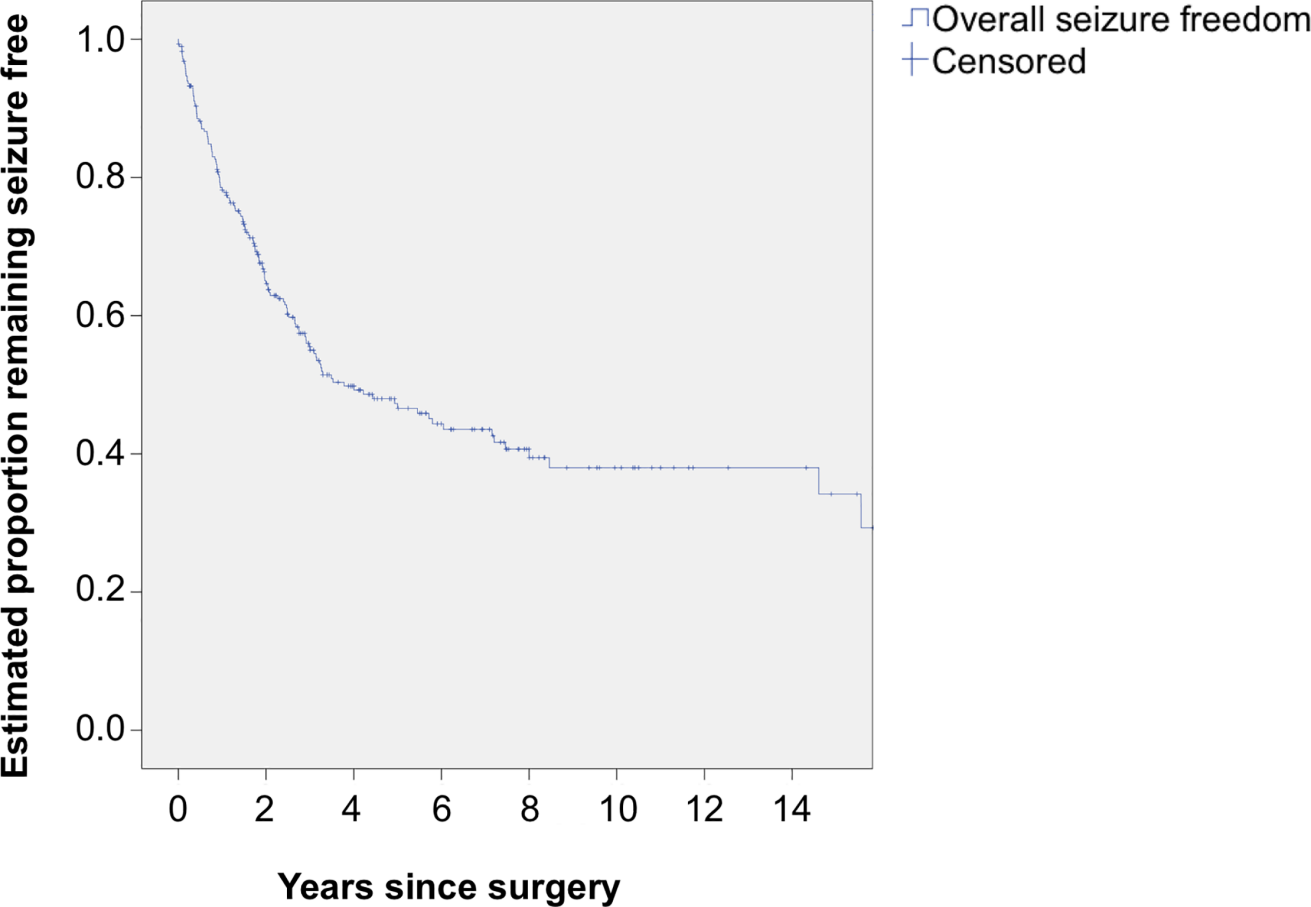
Kaplan-Meier analysis of time to first seizure.

The overall mean number of years to remain seizure free after surgery was 8.4 years (95% CI 7.1–9.7) regardless of operative procedure. Further analysis showed that 47% (n = 133) (95% CI 40–58) remained seizure free at 5 years and 38% (n = 108) (95% CI 31–45) at 10 years after surgery (Fig 1).

### Seizure freedom and operation type

**Fig 2:**
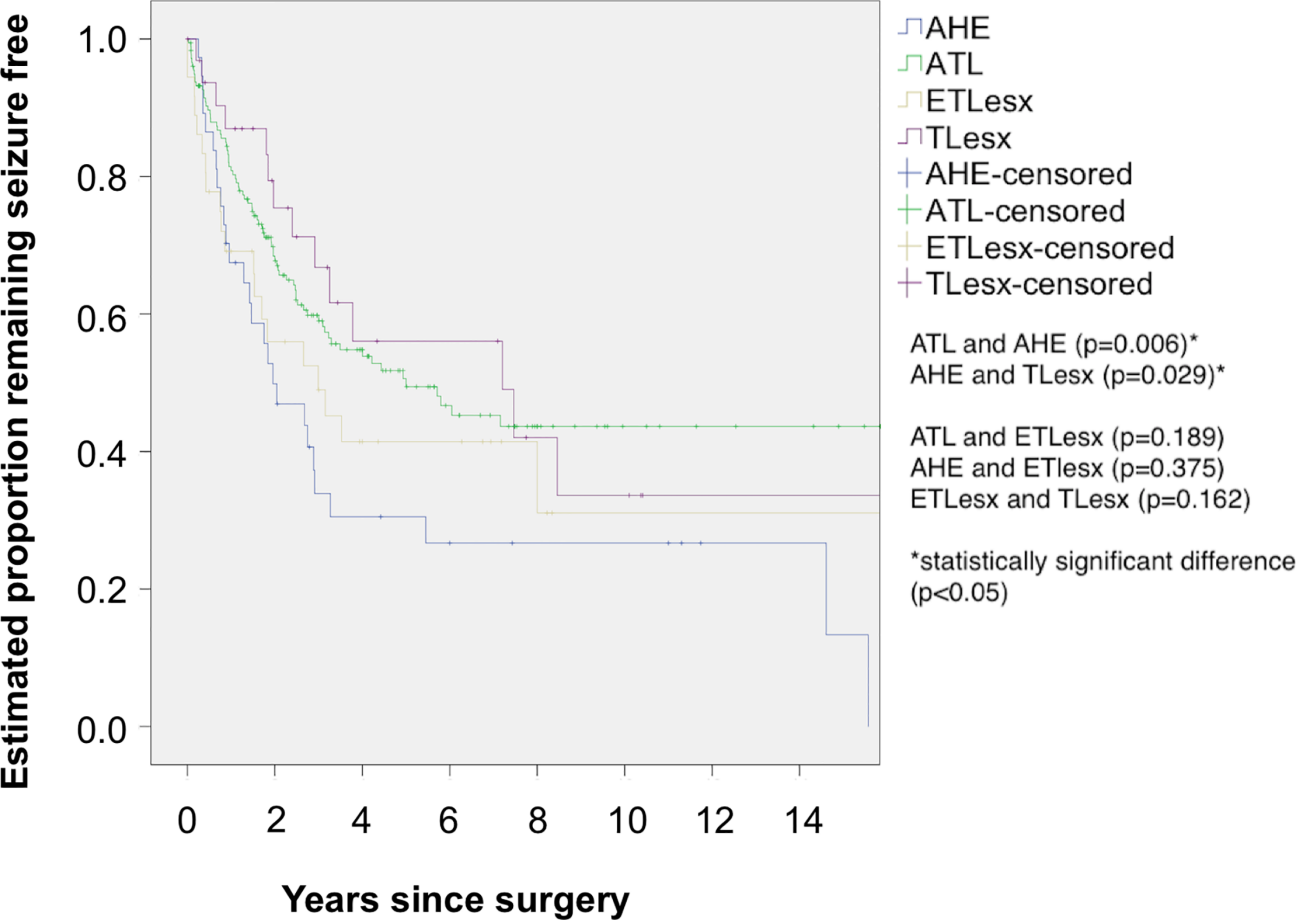
Kaplan-Meier analysis of time to first seizure by operative procedure. AHE = amygdalohippocampectomy, ATL = anterior temporal lobe resection, ETLesx = extra temporal lesionectomy, TLesx = temporal lesionectomy.

The mean number of years remaining seizure free was 9.9 years (95% CI 8.1–11.6) for ATL, 5.3 years (95% CI 3.1–7.4) for AHE, 6.8 years (95% CI 4.0–9.6) for ETLesx and 8.9 years (95% CI 5.5–12.3) for TLesx. There was a statistically significant difference between ATL and AHE (p = 0.006) (Log Rank (Mantel-Cox) test), and AHE and TLesx (p = 0.029) (Log Rank (Mantel-Cox) test). There was no statistically significant difference between ATL and ETLesx (p = 0.189) (Log Rank (Mantel-Cox) test), AHE and ETlesx (p = 0.375) (Log Rank (Mantel-Cox) test), ETLesx and TLesx (p = 0.162) (Log Rank (Mantel-Cox) test). Further analysis showed that after 5 years 49% (n = 87) (95% CI 41–57) who had ATL, 31% (n = 11) (95% CI 15–47) who had AHE, 42% (n = 15) (95% CI 25–59) who had ETLesx and 56% (n = 18) (95% CI 37–75) who had TLesx remained seizure free. At 10 years postoperatively, 44% (n = 78) (95% CI 35–53) who had ATL, 26% (n = 10) (95% CI 12–40) who had AHE, 31% (n = 11) (95% CI 9–53) who had an ETLesx and 34% (n = 11) (95% CI 10–58) who had TLesx remained seizure free (Fig 2).

### Seizure freedom and underlying pathology as determined by preoperative MRI

**Fig 3:**
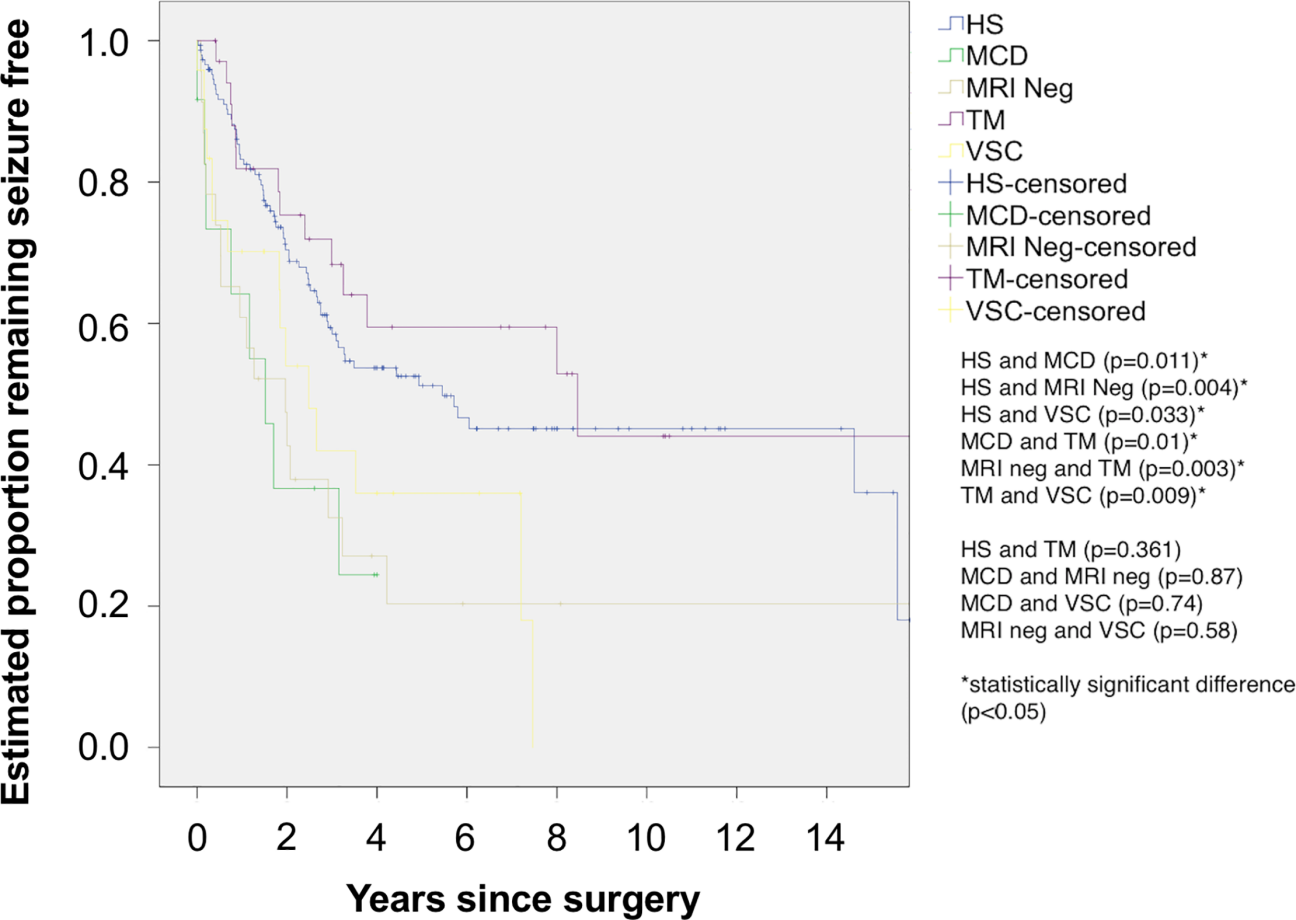
Kaplan-Meier analysis of time to first seizure by underlying pathology as determined by preoperative MRI. HS = hippocampal sclerosis, MCD = malformations of cortical development, MRI Neg = MRI negative, TM = tumour, VSC = vascular.

The mean number of years remaining seizure free was 8.2 years (95% CI 7.0–9.5) for HS, 1.9 years (95% CI 1.0–2.8) for MCD, 4.7 years (95% CI 1.8–8.0) for MRI neg, 10.2 years (95% CI 6.9–13.6) for TM and 3.5 years (95% CI 2.2–4.9) for VSC. There was a statistically significant difference between HS and MCD (p = 0.011) (Log Rank (Mantel-Cox) test), HS and MRI Neg (p = 0.004) (Log Rank (Mantel-Cox) test), HS and VSC (p = 0.033) (Log Rank (Mantel-Cox) test), MCD and TM (p = 0.01) (Log Rank (Mantel-Cox) test), MRI neg and TM (p = 0.003) (Log Rank (Mantel-Cox) test), TM and VSC (p = 0.009) (Log Rank (Mantel-Cox) test). There was no statistically significant difference between HS and TM (p = 0.361) (Log Rank (Mantel-Cox) test), MCD and MRI neg (p = 0.87) (Log Rank (Mantel-Cox) test), MCD and VSC (p = 0.74) (Log Rank (Mantel-Cox) test), MRI neg and VSC (p = 0.58) (Log Rank (Mantel-Cox) test). In the cohort with HS, 51% (n = 75) (95% CI 42–60) remained seizure free at 5 years and 44% (n = 65) (95% 35–53) at 10 years. For the MCD cohort 38% (n = 5) (95% CI 8–68) remained seizure free at 4 years 0% at 10 years. For the MRI neg cohort 20% (n = 4) (95% CI 2–38) remained seizure free at 5 years and 20% (n = 4) (95% CI 2–38) at 10 years. For the TM cohort 60% (n = 20) (95% CI 45–75) remained seizure free at 5 years and 44% (n = 15) (95% CI 21–67) at 10 years. For the VSC cohort, 36% (n = 8) (95% CI 14–58) remained seizure free at 5 years, 0% at 10 years (Fig 3).

### Changing underlying pathology

**Fig 4:**
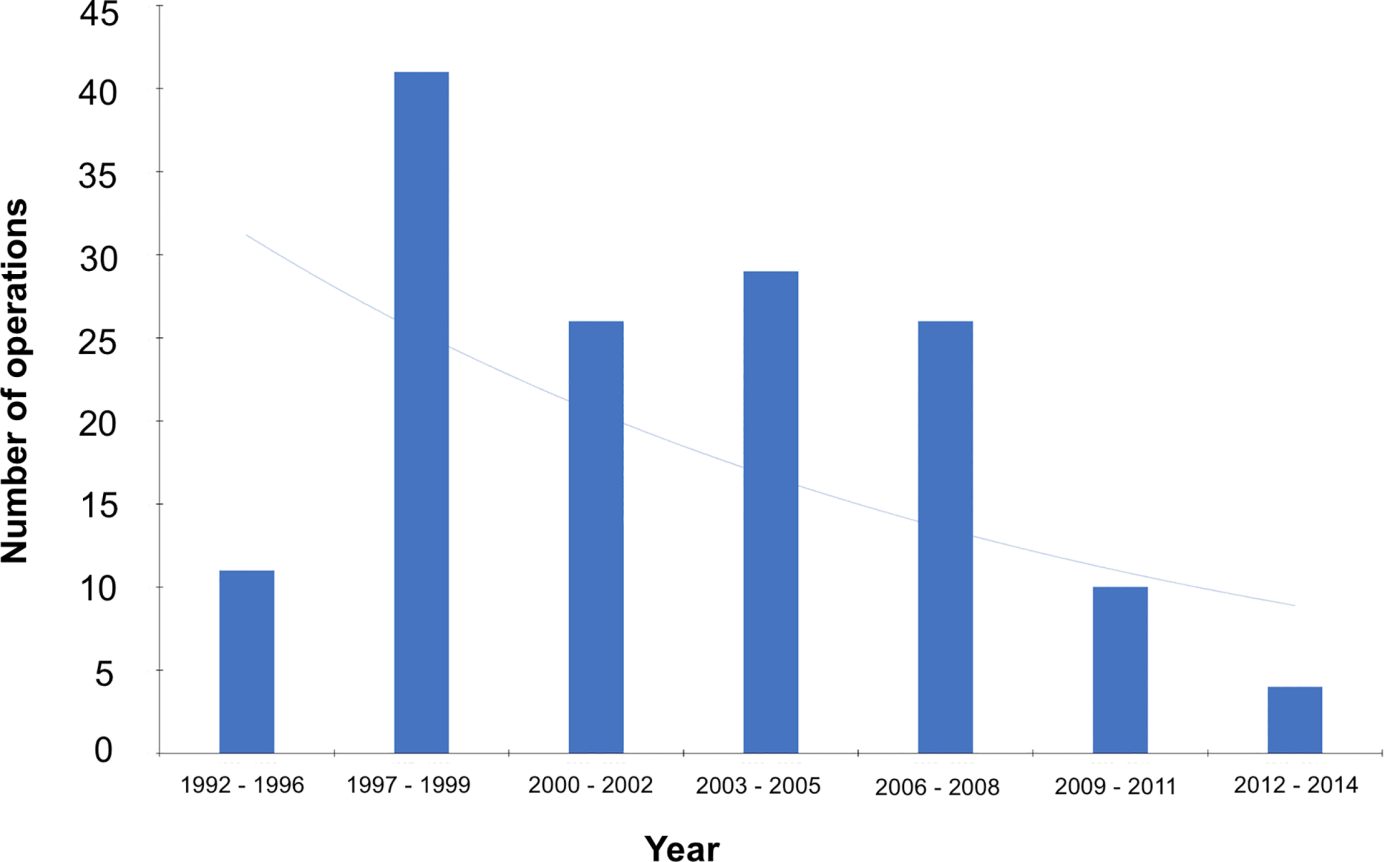
Number of surgical procedures performed for hippocampal sclerosis over time.

From the year 1997 onwards the number of operations performed for hippocampal sclerosis had decreased with 41 operations performed between the time period of 1997–1999 and four operations performed between 2012 and 2014 (Fig 4).

### Improved epilepsy (ILAE Class 4 or better)

**Fig 5:**
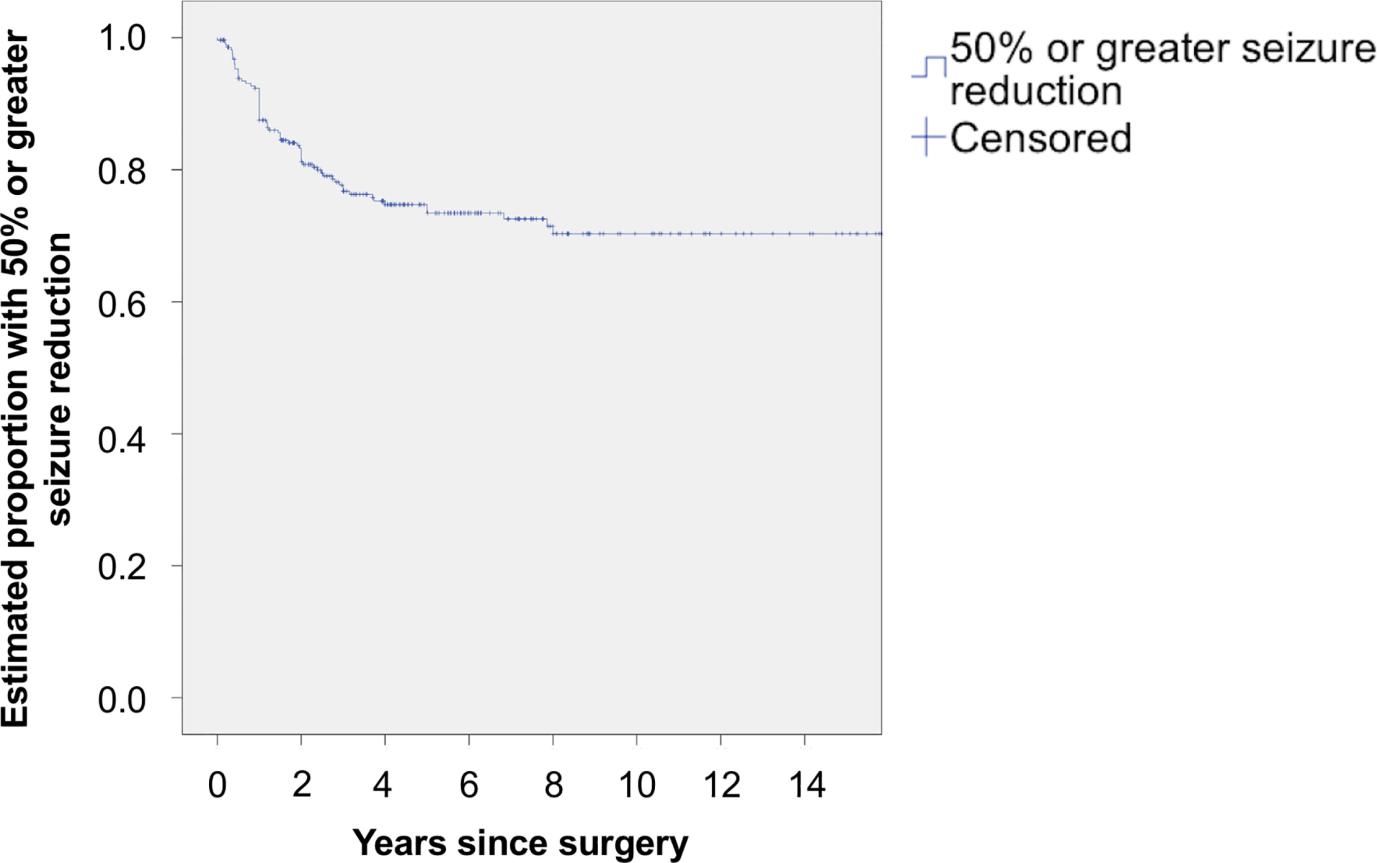
Kaplan-Meier analysis of estimated proportion with a 50% or greater seizure reduction.

Seventy four percent (n = 210) (95% CI 69–80) had a >50% seizure reduction at 5 years and 70% (n = 199) (95% CI 64–77) at 10 years (ILAE Class 1 to 4) (Fig 5).

## Discussion

The aim of this study was to determine long-term seizure outcomes after epilepsy surgery in a large cohort of patients from a dedicated neurosurgical hospital in the U.K. Our survival analysis estimated that 47% patients were free from debilitating seizures at 5 years and 38% at 10 years. These outcomes fall within the range of published long-term outcomes [9,14,16,17,22,28,32,37,45–52]. The reason for the heterogeneity in seizure outcomes are likely to be multifactorial. One reason may be due to how the authors defined seizure freedom and whether or not they included SPS in their definition. Another possible reason is the different post-surgical AED treatment and tapering methods used. The heterogeneity in the use of AEDs post epilepsy surgery is likely due to there being no consensus statement or robust evidence on the timeframes for when AEDs should be tapered or stopped post successful surgery. [53]. Furthermore, a study conducted on patients with epilepsy who were medically treated showed that how the possible risks are conveyed to the patient greatly influences their decision whether to taper or stop the AEDs [54]. The study used a computer based predictive model to counsel patients about their individual risk of seizure recurrence and the majority of patients opted to continue with AEDs. Therefore, the way the risks associated with stopping and tapering AEDs are conveyed could have a significant impact on the patient’s decision and subsequently add to the heterogeneity seen in seizure freedom rates in the literature.

Interestingly, the reported long-term outcomes of surgery in the modern MRI era is similar to the outcomes of surgical therapy for temporal lobe seizures in patients with non-tumour lesions prior to the advent of modern imaging. In a study that ranged from 1930 to 1967, 49% of patients were completely seizure free or seizure free after some postoperative attacks with a follow-up ranging from 2–36 years (median 8 years) [55]. Tellez-Zenteno [6] observed only a trend for improved long-term seizure outcomes for patients with temporal lobe epilepsy operated after 1980, compared to patients operated prior to 1980 before MRI became available. There can be no doubt that MRI has enabled the pre-surgical detection of important structural abnormalities such as hippocampal sclerosis [56], however, the advent of MRI has not necessarily translated into a significant improvement of long-term outcomes.

In our study the Kaplan-Meier curve, very similar to other studies, [9,32] started to flatten after an initial steep decline at about 4 years and flattened to almost a plateau at about 6 years, although late recurrences did occur. It is unclear if patients can be considered to be completely cured in the very long-term by epilepsy surgery or if the epilepsy is merely converted from a medical refractory to a medically treatable form and continue to require anti-epileptic medication. The reason for late recurrences is unclear. Interestingly, about 50% of patients who underwent supratentorial neurosurgery for a wide variety of reasons other than epilepsy also developed seizures within 5 years, suggesting the surgery itself could be the cause of seizure recurrence [57].

### Operation type and long-term outcome

In the present study, those who had an ATL had a better long-term outcome than those who had AHE. Patients with AHE had a very limited resection of amygdala and hippocampal head but not including the parahippocampal gyrus. Although a limited resection did not always preclude a good long-term outcome in individual cases, overall a better outcome was achieved with larger resections [58,59]. Studies suggest that removal of the parahippocampal gyrus improved outcomes in keeping with the hypothesis for an amplifier role of the parahippocampal gyrus [60]. A recent study by Mathon et al [61] shows similar seizure outcome between ATL and trans-superior temporal gyrus AHE. Another study by Keller et al [62] reports superior outcomes in patients who had a greater resection of the uncinate fasciculus which runs through the temporal stem. Therefore, we speculate that disconnection of the temporal stem may also be a factor.

In keeping with a large multi-centre study the outcome of medial temporal resections was not significantly different to neocortical resections [48]. The study by de Tisi et al [9] reported that patients who had an ATL had better seizure freedom rates compared to ETLesx. In our study the difference was not statistically significant.

### Underlying pathology and long-term outcome

Our results showed patients with hippocampal sclerosis had a significantly greater chance of long-term postoperative seizure freedom compared to MRI negative patients. Hippocampal sclerosis had been identified as the cause of epilepsy in some patients with epilepsy over 100 years ago [63] and is a well-known predictor of good outcome [48]. However, our study showed that the prevalence of hippocampal sclerosis was declining relative to the prevalence of other pathologies or no structural abnormality on MRI over the study period. The exact prevalence of hippocampal sclerosis amenable to epilepsy surgery in the general epilepsy population is unknown. Post mortem studies have demonstrated a high prevalence of hippocampal or Ammon’s horn sclerosis (66%) in selected patients [64]. In a large study 160 of 450 patients (35.5%) with chronic epilepsy had Ammon’s horn sclerosis. However, only 68 (15%) had a unilateral Ammon’s horn sclerosis. Of the unilateral cases, 54 had other (more widespread) additional pathologies including hemiatrophy, cystic defects, tumours, arteriosclerosis, trauma, leaving only 14 (3%) “ideal candidates” for surgery [65]. MRI studies mirrored the pathological studies. A high prevalence of hippocampal sclerosis (between 24% [66] to 32% [67]) was found in selected patients with epilepsy attending a tertiary referral centre. However, in unselected patients after a first seizure the prevalence of hippocampal sclerosis was only 5% [68]. Gowers commented in 1893 that overall “no greater significance can be ascribed to the induration of the Cornu Ammonis (pes hippocampi) [as a pathological substrate of epilepsy]” [69]. The data suggest that the surge in cases with hippocampal sclerosis was due to a backlog of patients. It is quite likely that in the future the mix of underlying pathologies will significantly change. Our results suggest that the outcomes of patients with normal MRI or vascular abnormalities are not as favourable as the outcomes of patients with hippocampal sclerosis. Further studies are required for malformations of cortical development but our relatively short-term data are not promising. Our results on outcome predictors can therefore not be automatically transferred to other, future patient groups with a different mix of underlying pathologies.

### Long-term outcome of non-seizure free patients

It is well known that epilepsy surgery is far superior to standard anti-epileptic drug treatment in selected patients in the short-term [4]. For obvious ethical reasons there is a lack of randomised long-term studies with non-surgical control groups. However, observational studies suggested that with conventional anti-epileptic drug treatment only 21% of patients, who were considered for surgery but for a range of reasons were not operated, entered remission and remained seizure free for an average of 2.5 years [70]. Our study showed the vast majority of patients who were not completely seizure free experienced at least a substantial and long lasting reduction in seizure frequency. Seventy percent of patients had at least a 50% reduction of seizure frequency 10 years after surgery. This finding is supported by Yoon et al [71] who found that half of the patients who relapsed after epilepsy surgery had at most one seizure per year. Epilepsy surgery therefore compared favourably to all new anti-epileptic drugs including Lamotrigine, Oxcarbazepine, Topiramate, Gabapentin [72] Levetiracetam, Remacemide, Zonisamide, [73] Eslicarbazepine, Retigabine, Carisbamate, Lacosamide, Brivaracetam, Perampanel, [74] and Cannabidiol [75]. Therefore, in patients who are refractory to conventional antiepileptic drugs and who are candidate for resective epilepsy surgery, surgery appears to be preferable to trying the latest anti-epileptic drug. Further studies are required to determine if a mere reduction in seizure frequency as opposed to complete seizure freedom still results in an improved quality of life.

#### Limitations

This study has several limitations. Firstly our study is skewed towards temporal lobe resections. Unfortunately, this is an inevitable since ATL resection is always the most common form of epilepsy surgery performed in large neurosurgical centres. Our study design is pragmatic and we included all possible cases, thus providing a real world perspective on epilepsy surgery. Furthermore, high impact studies have been published with their data skewed towards ATL resection. For example in the Lancet study by de Tisi et al[76], over 80% of their operations were ATL (497 out of 615).

Secondly, we did not investigate the issue of outcomes and subsequent post-operative death due to lack of data being available. For 12 out of 15 patients that had died, we did not have any data for the underlying cause of death. We acknowledge this is a major limiting factor since if the deaths were due to SUDEP, this would be indicative of surgical failure. Nevertheless, the principal aim of this study was to investigate the seizure outcomes after epilepsy surgery which has been achieved.

## Conclusion

Our study shows that surgery is effective in providing long-term seizure freedom in patients with refractory epilepsy; almost three quarters of patients had a greater than 50% seizure reduction in seizure frequency at five years and 70% had this reduction at 10 years. Almost half of all patients remained free from debilitating seizures at five years, and over a third of patients at 10 years. Hippocampal sclerosis and ATL was associated with positive long-term seizure outcome.

## Supporting information

S1 Table Clinical data on all patients. (XLSX)
